# Supplementary material for: Integrative experimental/computational approach establishes active cellular protrusion as the primary driving force of phagocytic spreading by immune cells
Source: PLoS Comput Biol. 2022 Aug 26;18(8):e1009937. doi: 10.1371/journal.pcbi.1009937 (PMC9455874; doi:10.1371/journal.pcbi.1009937)
Supplement: S1 Table — (PDF) [file pcbi.1009937.s007.pdf]

S1 Table. Modeling parameters

Parameter	Description	Value	Notes
τ_0	Resting cortical tension	0.01 mN/m	Matches experimental value used by Herant et al [1]
τ_{max}	Maximum cortical tension	~1 mN/m (see constitutive relation)	In a reasonable range for high tensions measured during neutrophil phagocytosis [2, 3]
μ	Effective cytoplasmic viscosity	200 Pa-s (passive) 1,660 Pa-s (active)	Matches experimentally measured values [4, 5]
κ_b	Effective membrane bending modulus	1×10^{-18} J	Matches experimentally measured values [6], much higher than values for RBCs or vesicles due to the neutrophil cortex
R_0	Initial cell radius	4.25 μm	Radius of a human neutrophil
σ_0	Adhesion stress constant	370 Pa	Together with the ligand density, this determines adhesion strength
$\rho_{IgG,max}$	Ligand density corresponding to 100% for Brownian Zipper model	10,000 IgG/μm^2	This density corresponds to about 600 $\mu J/m^2$, which exceeds values derived from other cases of cell spreading [7]
D_0	Zero adhesion force distance	50 nm	Relatively large distance required for mesoscopic model, as standard in other continuum models [8, 9]
$\sigma_{prot,max}$	Max. protrusion stress	3,500 Pa	Actin filaments growing in parallel can achieve forces above 1 nN per μm^2 (> 1 kPa) [10, 11]
s_0	Protrusive force range	0.8 μm	Controls how the protrusion stress decays along the membrane (Eq 8)
t_0	Characteristic time for decay of protrusion stress	66 s	Used for discrete adhesion model (Eq 13)
k_BT	Energy scale factor	4.11×10^{-21} J	Boltzmann constant (k_B) times room temperature (298 K), sets scale for membrane fluctuations (Eq 12) and ligand-receptor binding energy.
$\rho_{Fc\gamma R}$	FcγR density in the neutrophil membrane	1,470 μm^{-2}	Receptor density used in discrete ligand + discrete receptor simulations shown in Fig 8B, explained in Appendix F
D_{eff}	Effective FcγR diffusion coefficient	1×10^{-4} $\mu m^2/s$	Chosen value explained in Appendix F

References

1. Herant M, Lee CY, Dembo M, Heinrich V. Protrusive push versus enveloping embrace: computational model of phagocytosis predicts key regulatory role of cytoskeletal membrane anchors. PLoS Comput Biol. 2011;7(1):e1001068. doi: 10.1371/journal.pcbi.1001068. PubMed PMID: 21298079; PubMed Central PMCID: PMCPMC3029235.

2. Lee CY, Thompson GR, 3rd, Hastey CJ, Hodge GC, Lunetta JM, Pappagianis D, et al. Coccidioides Endospores and Spherules Draw Strong Chemotactic, Adhesive, and Phagocytic Responses by Individual Human Neutrophils. PLoS One. 2015;10(6):e0129522. doi: 10.1371/journal.pone.0129522. PubMed PMID: 26070210; PubMed Central PMCID: PMCPMC4466529.

3. Herant M, Heinrich V, Dembo M. Mechanics of neutrophil phagocytosis: behavior of the cortical tension. J Cell Sci. 2005;118(Pt 9):1789-97. doi: 10.1242/jcs.02275. PubMed PMID: 15827090.

4. Evans E, Yeung A. Apparent viscosity and cortical tension of blood granulocytes determined by micropipet aspiration. Biophys J. 1989;56(1):151-60. Epub 1989/07/01. doi: 10.1016/S0006-3495(89)82660-8. PubMed PMID: 2752085; PubMed Central PMCID: PMCPMC1280460.

5. Tran-Son-Tay R, Needham D, Yeung A, Hochmuth RM. Time-dependent recovery of passive neutrophils after large deformation. Biophys J. 1991;60(4):856-66. Epub 1991/10/01. doi: 10.1016/S0006-3495(91)82119-1. PubMed PMID: 1742456; PubMed Central PMCID: PMCPMC1260136.

6. Zhelev DV, Needham D, Hochmuth RM. Role of the membrane cortex in neutrophil deformation in small pipets. Biophys J. 1994;67(2):696-705. Epub 1994/08/01. doi: 10.1016/S0006-3495(94)80529-6. PubMed PMID: 7948682; PubMed Central PMCID: PMCPMC1225412.

7. Cuvelier D, Thery M, Chu YS, Dufour S, Thiery JP, Bornens M, et al. The universal dynamics of cell spreading. Curr Biol. 2007;17(8):694-9. Epub 2007/03/24. doi: 10.1016/j.cub.2007.02.058. PubMed PMID: 17379524.

8. Sukumaran S, Seifert U. Influence of shear flow on vesicles near a wall: A numerical study. Phys Rev E. 2001;64(1). doi: ARTN 011916

DOI 10.1103/PhysRevE.64.011916. PubMed PMID: WOS:000169907100090.

9. Etienne J, Duperray A. Initial dynamics of cell spreading are governed by dissipation in the actin cortex. Biophys J. 2011;101(3):611-21. Epub 2011/08/03. doi: 10.1016/j.bpj.2011.06.030. PubMed PMID: 21806929; PubMed Central PMCID: PMCPMC3145312.

10. Parekh SH, Chaudhuri O, Theriot JA, Fletcher DA. Loading history determines the velocity of actin-network growth. Nat Cell Biol. 2005;7(12):1219-23. Epub 2005/11/22. doi: 10.1038/ncb1336. PubMed PMID: 16299496.

11. Marcy Y, Prost J, Carlier MF, Sykes C. Forces generated during actin-based propulsion: a direct measurement by micromanipulation. Proc Natl Acad Sci U S A. 2004;101(16):5992-7. Epub 2004/04/14. doi: 10.1073/pnas.0307704101. PubMed PMID: 15079054; PubMed Central PMCID: PMCPMC395911.
